# Socioeconomic inequality in recommended childhood vaccinations in Germany: evidence from insurance claims and concentration index decomposition

**DOI:** 10.1186/s12916-026-05113-2

**Published:** 2026-08-04

**Authors:** Christopher Nussbaum, Leonie Sundmacher, Wiebke Schuettig

**Affiliations:** 1 Chair of Health Economics, School of Medicine and Health, Technical University of Munich, Am Olympiacampus 11, 80809 Munich, Germany; 2 Munich Center for Health Economics and Policy (M-CHEP), Am Olympiacampus 11, 80809 Munich, Germany

**Keywords:** Concentration index, Decomposition, Vaccination, Socioeconomic status, Insurance claims data, Pediatric health data

## Abstract

**Background:**

Despite well-established childhood immunization programs in many high-income countries, vaccination series are often delayed or not completed. Gaps in vaccination coverage are more common among children from socioeconomically disadvantaged households, among whom they are associated with higher rates of related hospitalizations. We aimed to assess the extent of these gaps in Germany, as well as the presence of socioeconomic inequality and the factors accounting for it.

**Methods:**

Using health insurance claims data from Techniker Krankenkasse for 2016–2019, we examined vaccination coverage for a comprehensive set of STIKO-recommended childhood vaccinations in six birth-year cohorts (2011–2018; approximately 430,000 children; 2.5 million recommended vaccinations). Coverage was defined as completion of the recommended series with all doses administered within the recommended age window. Socioeconomic inequality was quantified using the Erreygers-corrected concentration index and decomposed into contributions from factors such as parental education and travel time to the treating physician.

**Results:**

Approximately 80% of vaccination series in the sample were complete and administered within the recommended age window. Vaccination coverage showed a small but statistically significant socioeconomic gradient (concentration index = 0.024, 95% confidence interval: 0.023 to 0.025) favoring children from higher socioeconomic households. In the decomposition analysis, provider type accounted for the largest share of this gradient, with lower completion rates and timeliness among children whose outpatient care was mainly delivered by general practitioners compared with pediatricians. Parental education contributed to a lesser extent, and travel time contributed little to the observed inequality.

**Conclusions:**

Childhood vaccination coverage in Germany remains below national targets and shows a small socioeconomic gradient. The gradient appears to be less strongly related to logistical access barriers than to differences in how vaccinations are delivered in outpatient care outside pediatric settings. These provider-type differences in vaccination delivery may therefore be more relevant for narrowing the observed gradient than further reductions in travel time.

**Supplementary Information:**

The online version contains supplementary material available at 10.1186/s12916-026-05113-2.

## Background

Outbreaks of vaccine-preventable diseases are increasing worldwide, with some infections re-emerging in places where they had been kept under control [1]. In many high-income countries, including those with well-established childhood vaccination programs, immunization targets are missed because recommended vaccinations are delayed or not completed [2]. These shortfalls are not evenly distributed across populations. Higher rates of vaccine-preventable disease-related hospitalizations have been observed among individuals from lower socioeconomic backgrounds [3, 4]. This is particularly concerning for children and adolescents, who have limited control over their own health, its determinants, and the longer-term consequences [5, 6].

Research on differences in childhood vaccination coverage has mostly focused on macro-level features of health systems, such as the organization of primary care and preventive services, where many high-income countries already perform well [7, 8]. At the same time, studies have identified a range of individual- and household-level factors associated with children’s and adolescents’ vaccination status within the same health care system. Although findings vary across settings and vaccine types, lower coverage has been linked to residence in isolated or deprived areas [9, 10], a higher number of siblings [9, 11], and a recent family history of migration [11]. In line with Andersen’s health service use model, higher vaccination coverage is observed among children from households with higher socioeconomic status (SES), commonly defined by parental income, educational attainment, or occupation [10, 12, 13]. Parental beliefs and attitudes, including vaccine hesitancy, represent an additional factor and are closely linked to several of these characteristics [12, 14–17].

Much of the existing literature is based on relatively small survey samples and self-reported information, which limits objective population-level assessment. Health insurance claims provide a valuable alternative source of data for studying childhood immunization patterns at scale [18, 19]. At the same time, evidence on the role of area-level factors and how these vary by SES is scarce despite their relevance for understanding socioeconomic differences in vaccination coverage [8]. In Germany, socioeconomic differences in childhood and adolescent immunization are not well characterized. Identifying such inequality, however, is only the first step; it is also important to examine which factors account for the observed socioeconomic gradient in coverage to develop targeted interventions.

In the present study, we aimed to assess the extent of gaps in childhood vaccination coverage in Germany, to quantify socioeconomic inequality using the concentration index (CI), and to examine how individual- and area-level factors contribute to this inequality [20]. To date, most applications of the CI in this area have focused on low- and middle-income countries. Building on evidence from high-income countries with universal health coverage, we focused in particular on parental education and geographic access to care as factors that may account for part of the socioeconomic inequality assessed in this study [12]. Lower parental education has been linked to lower health literacy, which may affect awareness of the recommended vaccine schedules and the importance of timely immunization [21, 22]. In addition, economic constraints and competing priorities may increase the perceived burden of travel to vaccination providers, further contributing to lower uptake among families with lower SES [8, 23].

## Methods

### Data sources

We used health insurance claims data from Techniker Krankenkasse (TK), the largest statutory health insurer in Germany. TK insured approximately 10 million people across all German states in 2016, corresponding to about 12% of the total population in Germany [24]. As a nationwide insurer, TK covers individuals in all federal states, and the regional distribution of its insured population broadly follows the overall population distribution, although the proportion of TK-insured individuals varies between states [24]. At that time, around 120 sickness funds operated in Germany; this number has since declined to 93 in 2026 [25]. The data set covered insured individuals from the birth-year cohorts 1995 through 2019 during the pre-COVID study period (2016–2019). In Germany, statutory health insurance provides family coverage, under which children are insured through a parent’s insurance. Children insured through this family insurance were linked to the policy-holding parent, enabling analyses of parental socioeconomic status (SES). Children’s SES therefore reflected the socioeconomic background of the policy-holding parent only. As information on the other parent was not available, household-level SES could not be defined. We provide additional details on the selection of socioeconomic variables later in this section. The claims data include comprehensive information on reimbursable outpatient procedures and diagnoses, as well as on treating physicians and their medical specialties. In addition, we linked regional data based on the child’s place of residence from the Federal Office for Building and Regional Planning, as well as from Schang et al. (2017) [26, 27].

### Covered vaccinations

In Germany, healthcare is organized through a system of near-universal coverage, in which almost all residents have health insurance, predominantly through statutory health insurance and, to a lesser extent, private health insurance. Under this system, statutory health insurers are required to cover, free of charge, all vaccinations recommended by the Standing Committee on Vaccination (STIKO) at the Robert Koch Institute (RKI). For the period from 2016 to 2019, we included the following vaccinations recommended for children and adolescents (up to 17 years): rotavirus, tetanus, diphtheria, pertussis, *Haemophilus influenzae* type B, poliomyelitis, hepatitis B, pneumococcal disease, measles, mumps, rubella, varicella, and meningococcal C [28]. We did not include human papillomavirus vaccination because the recommended administration window (ages 9–14) extended beyond the available observation period, so vaccination status could not be determined reliably in our sample. Changes to the STIKO vaccination schedule during the observation period did not affect our analysis [29–31].

### Study population

We defined eligible birth cohorts based on the recommended administration window for each vaccine. We included a given cohort–vaccine combination if the recommended vaccination period fell within the available observation period. For example, for the 2018 birth cohort, the rotavirus series (2–3 doses) is recommended by the age of four months. This window overlapped with 2018–2019, which fell within our observation period, so rotavirus vaccination was assessed for this cohort. Exact birth dates were not available (only birth year), so the observation period could extend beyond the recommended vaccination window for some cohort–vaccine combinations.

For the final study population, we excluded children who were living abroad, died during the observation period, or did not have continuous insurance coverage with TK. We also excluded children whose parental occupation or education data were missing. Further details on missingness and sample selection are provided in the Additional file 1: Sect.  1.

### Outcome definition

The outcome was vaccination coverage, which we defined, in line with prior studies, as a binary indicator coded as “fully vaccinated” if the vaccination series was complete and administered within the recommended time window [32]. Vaccinations were identified using codes from the Association of Statutory Health Insurance Physicians, following RKI guidelines (see Additional file 1: Sect. 2) [18, 33, 34].

### Socioeconomic status

In the absence of income data, we used the occupational status of the policy-holding parent as the socioeconomic ranking variable, which has been shown to be associated with childhood vaccination status [10]. In line with previous literature, we did not construct a composite SES index in order to keep the ranking measure transparent and interpretable [12]. We classified occupational status into five ordered categories based on the classification of the German Federal Employment Agency: (0) unemployed, (1) unskilled or semi-skilled activities, (2) specialist activities, (3) complex specialist activities, and (4) highly complex activities [35]. These categories reflect increasing levels of job complexity and qualification. The occupational categories are based on the occupation code derived from employer reporting for socially insured employment. As this reporting system does not systematically cover self-employed individuals, they may not be fully or consistently represented in this variable.

### Statistical methods

We calculated the CI to quantify socioeconomic inequality in vaccination coverage and plotted the corresponding concentration curve. The curve shows the cumulative share of the population ranked by SES (x-axis) against the cumulative share of the vaccination outcome (y-axis) [36–38]. The CI is defined as twice the area between the concentration curve and the 45-degree line of equality and ranges from − 1 to 1 [36, 37]. It can be expressed as twice the covariance between the outcome and the fractional rank, divided by the mean of the outcome. Positive values indicate that coverage is concentrated among higher-SES groups, whereas negative values indicate concentration among lower-SES groups [36–38]. A value of zero indicates equality [36–39]. The magnitude of the CI reflects the extent of inequality and can be interpreted (in percentage terms) as the redistribution of the outcome from higher- to lower-SES groups that would be required to achieve equality [40].

Because vaccination status is a bounded variable, we used the Erreygers-corrected concentration index, which rescales the CI to account for the bounds of the outcome and expresses inequality in absolute terms [20, 41–46]. Alternative corrections exist, including the Wagstaff normalization, which reflect different normative perspectives on inequality in bounded variables [47]. In particular, the Erreygers correction corresponds to an absolute inequality perspective and satisfies desirable properties such as mirror and level independence [47]. Given our interest in absolute differences in vaccination uptake across socioeconomic groups, we considered the Erreygers correction more appropriate. Because the socioeconomic ranking variable is ordinal, we applied the extended fractional rank method to handle tied observations and obtain stable CI estimates [48].

We then decomposed the CI to assess how specific factors contributed to the observed socioeconomic inequality in vaccination coverage and to quantify their relative contributions [38]. The decomposition expresses the CI as the sum of the contributions of individual covariates and a residual component. A factor contributes to inequality when it is unevenly distributed across the socioeconomic ranking (i.e., it has a nonzero CI) and is associated with vaccination status after adjustment for the other variables included in the model (i.e., it has a nonzero estimated coefficient) [49]. We estimated these associations using a generalized linear model with a logit link. Because the decomposition is formally derived for linear models, applying it to nonlinear models relies on a linear approximation, whereby the decomposition is based on the model’s predicted outcome values rather than a direct interpretation of the estimated log-odds coefficients. This approach is common in applied studies using logistic regression for binary outcomes [50, 51]. An alternative approach is to estimate a generalized linear model with a binomial distribution and identity link, which preserves the linear decomposition property and yields results that do not depend on the choice of reference groups [52].

The percentage contributions of the factors sum to the explained portion of the overall CI, and the residual captures the unexplained component. It was calculated as the difference between the overall CI and the sum of the explained contributions [38]. A positive percentage contribution indicates that the factor contributes to a greater concentration of vaccination coverage among higher socioeconomic groups. An equal distribution of this factor across the socioeconomic spectrum would reduce the overall inequality in vaccination coverage, as measured by the CI, by the corresponding share of the CI [40].

Additional mathematical details, including the formal decomposition equations and their extensions to the Erreygers-corrected CI, are provided in the Additional file 1: Sect.  3.

### Covariates

We included the following covariates to examine their contribution to socioeconomic inequality in vaccination coverage, with a particular focus on parental education and travel time to the treating physician.

We operationalized educational attainment as a categorical variable using slightly adjusted CASMIN (Comparative Analysis of Social Mobility in Industrial Nations) levels adapted for Germany: (1a) no school-leaving qualification, (1b) lower secondary school certificate without vocational training, (1c) lower secondary school certificate with vocational training, (2b) intermediate school certificate without vocational training, (2a) intermediate school certificate with vocational training, (2c_gen) higher education entrance qualification without vocational training, (2c_voc) higher education entrance qualification with vocational training, (3a) university of applied sciences degree, (3b) university degree, and (3c) doctorate/PhD [53]. Coding details are provided in the Additional file 1: Sect. 2. Because this classification does not map one-to-one onto occupational categories, we considered education and occupation as separate measures. Parental education and occupation were moderately correlated (*r* = 0.55; see Additional file 1: Table S1), indicating that they capture related but distinct dimensions of SES. Parental education has been associated with preventive care use and vaccination uptake in children, although the results are mixed [54–58].

We obtained average travel times to the treating physician from Schang et al. (2017). These estimates are based on nationwide statutory health insurance claims and represent realized travel times by private car between the municipality of residence and the municipality of the physician actually visited. Travel times were calculated using a road-network model based on average travel speeds and therefore reflect realized access rather than travel to the nearest provider [26]. Longer travel times have been linked to lower or delayed vaccination uptake [57, 59] and, more generally, to lower use of preventive care in rural areas [55]. We therefore included travel time as an explicit access measure rather than relying on rural–urban categories alone [56].

We considered several individual- and area-level covariates based on factors known to be associated with childhood vaccination coverage. Adjustment for these covariates was intended to reduce confounding in the associations between the main explanatory variables and vaccination status, as several factors may be related to both. Adjustment for health care need was not required because the vaccinations we examined are recommended for all children in the included birth-year cohorts and medical contraindications are rare, particularly given the availability of allergen-free vaccine formulations [60, 61].

At the individual level, covariates were the child’s sex, the main type of health care provider (HCP) involved in his or her outpatient care, place of residence, and recent history of migration in the family. Prior studies report mixed evidence regarding the association between a child’s sex and vaccination uptake [10, 56, 62, 63]. For place of residence, we considered urban versus rural residence and residence in a state in eastern Germany, both of which have been linked to vaccination status in earlier work [9, 11]. In the latter case, historical differences in health systems and the strong prioritization of immunization during the socialist period may continue to affect vaccination-related attitudes and practices. A recent history of migration to Germany may affect a family’s ability to navigate the health care system and has been linked to lower preventive care use [55] and lower vaccination coverage [11, 57, 58, 63–65]. We defined recent migration history as the policy-holding parent not having German citizenship. We coded the main HCP type as a categorical variable with four categories: general practitioner (GP), pediatrician, specialist care only, and no HCP (i.e., no contact with the health care system). Children who visited both GPs and pediatricians were assigned to the provider type that accounted for the majority of claims. Those who saw neither but had specialist visits were classified as receiving specialist care only. Previous studies have reported higher vaccination rates among children receiving care from pediatricians [9, 66].

Area-level covariates were deprivation, child poverty, daycare attendance, and pediatrician density. We obtained deprivation data from the RKI and the remaining measures from the Federal Office for Building and Regional Planning [27, 67]. All area-level variables were z-standardized for inclusion in the regression models.

We measured deprivation using the German Index of Socioeconomic Deprivation (GISD), which captures multiple dimensions of socioeconomic disadvantage. Previous research indicates that children living in more deprived areas are less likely to be fully vaccinated [10, 63]. Child poverty was defined as the number of children under the age of 15 years who receive means-tested social benefits under Germany’s SGB II scheme, expressed as a proportion of all children in the same age group within the area. Whereas the GISD reflects overall deprivation, this indicator specifically captures households reliant on social assistance, which may face barriers such as difficulties attending health care appointments. Area-level child poverty captures the socioeconomic context of the area of residence and is conceptually distinct from individual-level SES. In our data, area-level child poverty was only weakly correlated with both parental education and parental occupation (see Additional file 1: Table S1), indicating that it reflects a separate contextual dimension. Area-level variables were included as contextual covariates in a single-level model rather than within an explicit multilevel framework. Because the objective of our analysis was to quantify the contributions of both individual- and area-level characteristics to socioeconomic inequality in vaccination coverage, we used a single-level decomposition framework. Consequently, the estimated associations should be interpreted as describing correlations between contextual characteristics and vaccination coverage rather than as independent contextual effects after accounting for within-area clustering. Daycare attendance was defined as the proportion of children younger than three years enrolled in daycare within the corresponding age group. Higher daycare attendance has been associated with higher vaccination completion in previous studies, possibly reflecting changes in parental risk perception once children enter group care and the influence of mandatory pre-enrollment vaccination counseling under German law [56, 59]. Pediatrician density was measured as the number of pediatricians per 10,000 children younger than 15 years and served as an additional indicator of health care access [8]. Based on preliminary analyses, we excluded place of residence, pediatrician density, and the regional share of children in poverty from the final model due to multicollinearity with other covariates.

We calculated descriptive statistics to compare differences between children who were fully vaccinated and those who were not. Group differences were assessed using standardized mean differences, chi-square tests for categorical variables, and ANOVA for continuous variables. Statistical significance was assessed at the 5% level.

We conducted sensitivity analyses including normalizations of the CI, alternative ranking variables, alternative outcome definitions, and alternative sets of covariates.

All analyses were performed in R, including the rineq package.

## Results

### Population characteristics

Based on our inclusion criteria, the final sample comprised six birth-year cohorts (2011, 2012, 2015, 2016, 2017, and 2018) and 34 cohort–vaccine combinations. For the 2011 and 2012 cohorts, we assessed receipt of the first booster dose of tetanus, diphtheria, and pertussis. For the 2015 cohort, we examined vaccination against meningococcal C. For the 2016 and 2017 cohorts, we evaluated uptake of the full set of recommended early childhood vaccinations (rotavirus, tetanus, diphtheria, pertussis, *Haemophilus influenzae* type B, poliomyelitis, hepatitis B, pneumococcal disease, measles, mumps, rubella, varicella, and meningococcal C). For the 2018 cohort, we assessed the rotavirus series. Because children could be eligible for more than one vaccine during the study period, they could contribute more than one person–vaccine observation. In total, the data set comprised 2,513,033 person–vaccine observations across 431,427 children. Table 1 summarizes sample characteristics at the person–vaccine level.

**Table 1:** Summary statistics

	Total	Full vaccination	Partial/No vaccination	*p*-value	Standardized mean differences
Vaccination cases (person-vaccine pairs)	*n* = 2,513,033	*n* = 2,004,571	*n* = 508,462		
**Year of birth**				< 0.0001	0.320
2011	7.5%	6.5%	11.7%		
2012	8.1%	7.1%	11.9%		
2015	3.0%	3.1%	2.5%		
2016	39.5%	40.5%	35.5%		
2017	38.9%	40.4%	33.2%		
2018	3.0%	2.3%	5.4%		
**Place of residence: Urban**	79.1%	79.5%	77.4%	< 0.0001	0.051
**Place of residence: Eastern Germany**	18.0%	18.0%	18.0%	0.6513	0.001
**Sex (female)**	48.8%	48.8%	48.5%	< 0.0001	0.006
**Recent family history of migration**	8.1%	8.2%	8.1%	0.0151	0.004
**Parental education (CASMIN)**				< 0.0001	0.058
CASMIN 1a: No school-leaving qualification	1.1%	1.0%	1.2%		
CASMIN 1b: Lower secondary school certificate without vocational training	1.4%	1.3%	1.6%		
CASMIN 1c: Lower secondary school certificate with vocational training	12.0%	12.0%	12.1%		
CASMIN 2b: Intermediate school certificate without vocational training	1.5%	1.4%	1.7%		
CASMIN 2a: Intermediate school certificate with vocational training	21.2%	21.3%	20.8%		
CASMIN 2c_gen: Higher education entrance qualification without vocational training	3.4%	3.3%	3.9%		
CASMIN 2c_voc: Higher education entrance qualification with vocational training	17.2%	17.3%	16.8%		
CASMIN 3a: University of Applied Sciences degree	6.1%	6.1%	6.2%		
CASMIN 3b: University degree	32.6%	32.6%	32.6%		
CASMIN 3c: Doctorate/PhD	3.5%	3.6%	3.1%		
**Parental occupation**				< 0.0001	0.083
Unemployed	3.5%	3.3%	4.6%		
un-/semi-skilled activities	3.4%	3.3%	3.8%		
specialist activities	43.8%	44.3%	42.0%		
complex specialist activities	19.2%	19.1%	19.6%		
highly complex activities	30.0%	30.0%	29.9%		
**Main physician**				< 0.0001	0.327
None	0.1%	0.0%	0.6%		
General practitioner	5.8%	4.1%	12.4%		
Pediatrician	93.6%	95.5%	86.4%		
Specialist	0.5%	0.4%	0.6%		
**Travel time to treating physician**,** average (in minutes)**	14.46	14.39	14.72	< 0.0001	0.043
**Pediatrician density (Pediatricians per 10.000 children under 15)**,** average**	5.27	5.27	5.25	< 0.0001	0.018
**Deprivation score (GISD)**,** average**	0.39	0.39	0.38	< 0.0001	0.047
**Regional share of children in poverty (recipients of SGB II benefits under the age of 15 per 100 inhabitants under the age of 15)**,** average**	15.4%	15.5%	14.7%	< 0.0001	0.107
**Regional share of children under three in daycare**,** average**	33.9%	33.9%	33.5%	< 0.0001	0.047

Note: To evaluate the statistical significance of differences between groups, we calculated p-values using Chi-square tests for categorical variables and ANOVA tests for continuous variables. Standardized mean differences are reported to quantify the magnitude of group differences independent of sample size. Cohorts 2013 and 2014 were excluded because parts of the vaccination administration windows for vaccines recommended for these birth cohorts fall outside the available observation period, preventing complete assessment of vaccination status

Approximately 80% of the recommended vaccinations in our sample met the study definition of being both complete and administered within the recommended age window. The German National Immunization Plan sets target coverage levels of greater than 95% for measles, mumps, rubella, hepatitis B, and pertussis, and greater than 90% for booster doses against diphtheria, tetanus, pertussis, and poliomyelitis [68]. These targets are consistent with the vaccination levels of approximately 85% to 95% that are generally cited as necessary to achieve disease elimination (and, for some infections, eradication) [68], but are notably higher than the coverage observed in our sample. In international comparisons, Germany tends to perform slightly below the United States (US) and around the European Union (EU) average for several routine childhood vaccinations [69]. These comparisons should be interpreted cautiously because vaccination schedules, age definitions, timeliness criteria, and data sources differ across countries.

Across all person–vaccine observations, 48.8% of the children were female, 79.1% lived in urban districts, 18.0% resided in eastern Germany, and 8.1% had a recent family history of migration. Female children and those in eastern Germany were broadly representative of the overall German population, whereas urban districts were slightly overrepresented (70%) and children with a recent family history of migration were slightly underrepresented (12%) [27, 70]. The most common parental education categories were 3b (master’s degree or equivalent, 32.6%), 2a (intermediate school certificate with vocational training, 21.2%), and category 2c_voc (higher education entrance qualification with vocational training, 17.2%). Overall, 3.5% of observations related to children whose policy-holding parent was unemployed (4.6% when each child is counted only once), which is broadly consistent with the national unemployment rate in Germany of about 5% to 6% between 2016 and 2019 [71]. In addition, 43.8% of observations related to policy-holding parents working in specialist occupations (the second lowest of the four complexity levels). Parental occupation in the analysis sample was similar to that among children with available socioeconomic data in the full TK data set. Based on the type of provider accounting for the largest share of each child’s outpatient contacts, 93.6% were primarily seen by pediatricians and 5.8% by GPs. The mean travel time to the treating physician was 14.5 min, and pediatrician density was 5.27 per 10,000 children younger than 15 years.

All variables except residence in eastern Germany differed statistically significantly between fully vaccinated and partially/not vaccinated observations in unadjusted comparisons. However, standardized mean differences indicate that most of these differences were small in magnitude, with larger differences observed for provider type and year of birth (Table 1).

Differences by birth cohort were particularly pronounced: the 2011 and 2012 cohorts were more common in the partial/no vaccination group (11.7% vs. 6.5%), whereas the 2016 and 2017 cohorts were more common in the fully vaccinated group (2016: 40.5% vs. 35.5%; 2017: 40.4% vs. 33.2%), which is consistent with a lower uptake of booster doses. Physician type also differed: GP-led care was more common in the partial/no vaccination group (12.4% vs. 4.1%), whereas pediatrician-led care was more common in the fully vaccinated group (95.5% vs. 86.4%). Parental education and parental unemployment also differed between the groups, but the absolute differences across categories were modest.

Vaccination coverage ranged from 73.5% in the lowest occupational group to 79.8% in the highest occupational group, with the highest coverage observed in the middle category (80.6%), corresponding to an absolute difference of 6.3% points between the lowest and highest groups.

### Concentration index

**Fig. 1:**
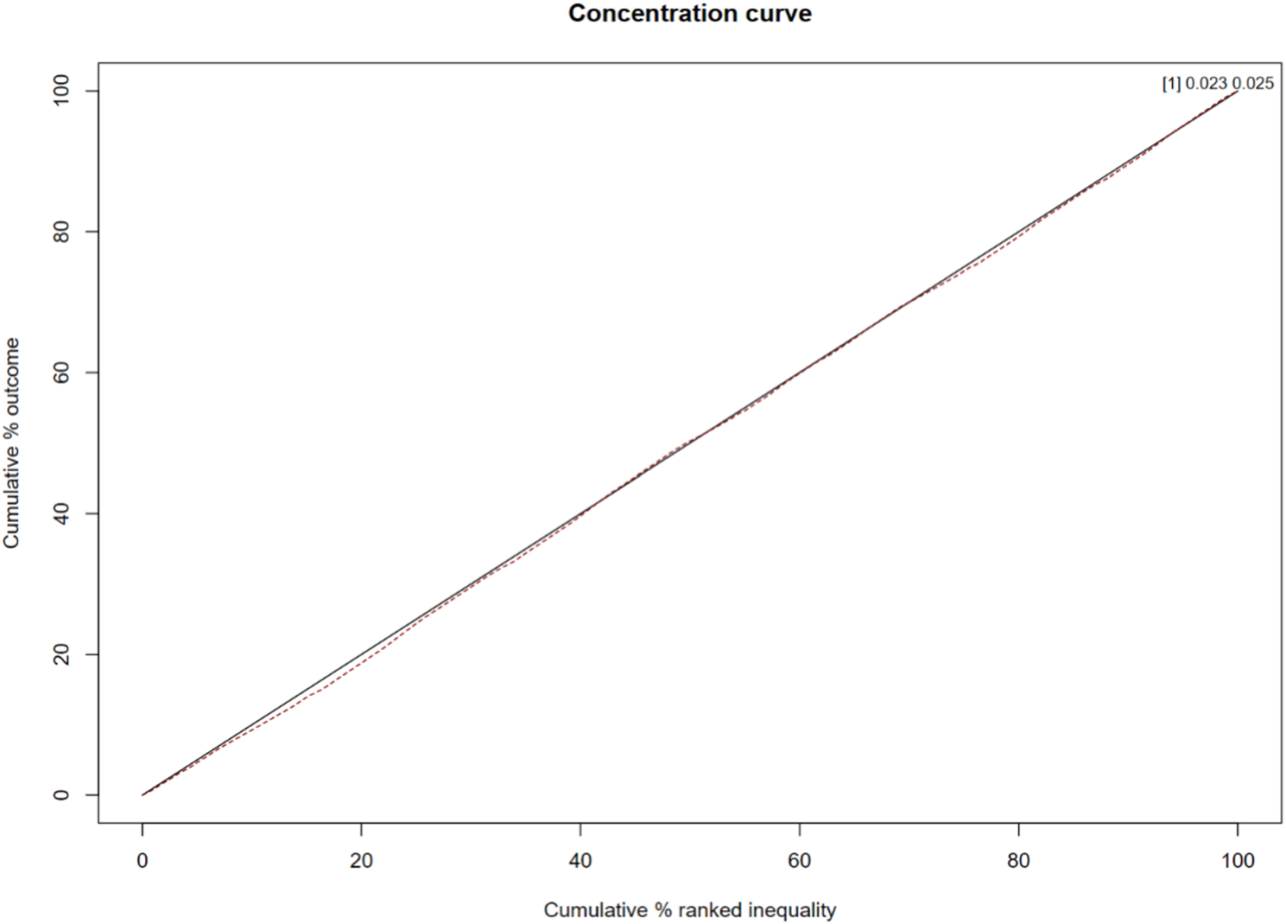
Concentration curve

The CI was 0.024 (95% confidence interval: 0.023 to 0.025), indicating a small but statistically significant concentration of full vaccination among children from higher socioeconomic households. The corresponding concentration curve is shown in Fig. 1. In stratified analyses, socioeconomic inequality was more pronounced for primary-series vaccinations (CI: 0.016; 95% confidence interval: 0.013 to 0.19) than for booster vaccinations (CI: 0.002; 95% confidence interval 0.000 to 0.003), where inequality was negligible.

### Decomposition

**Fig. 2:**
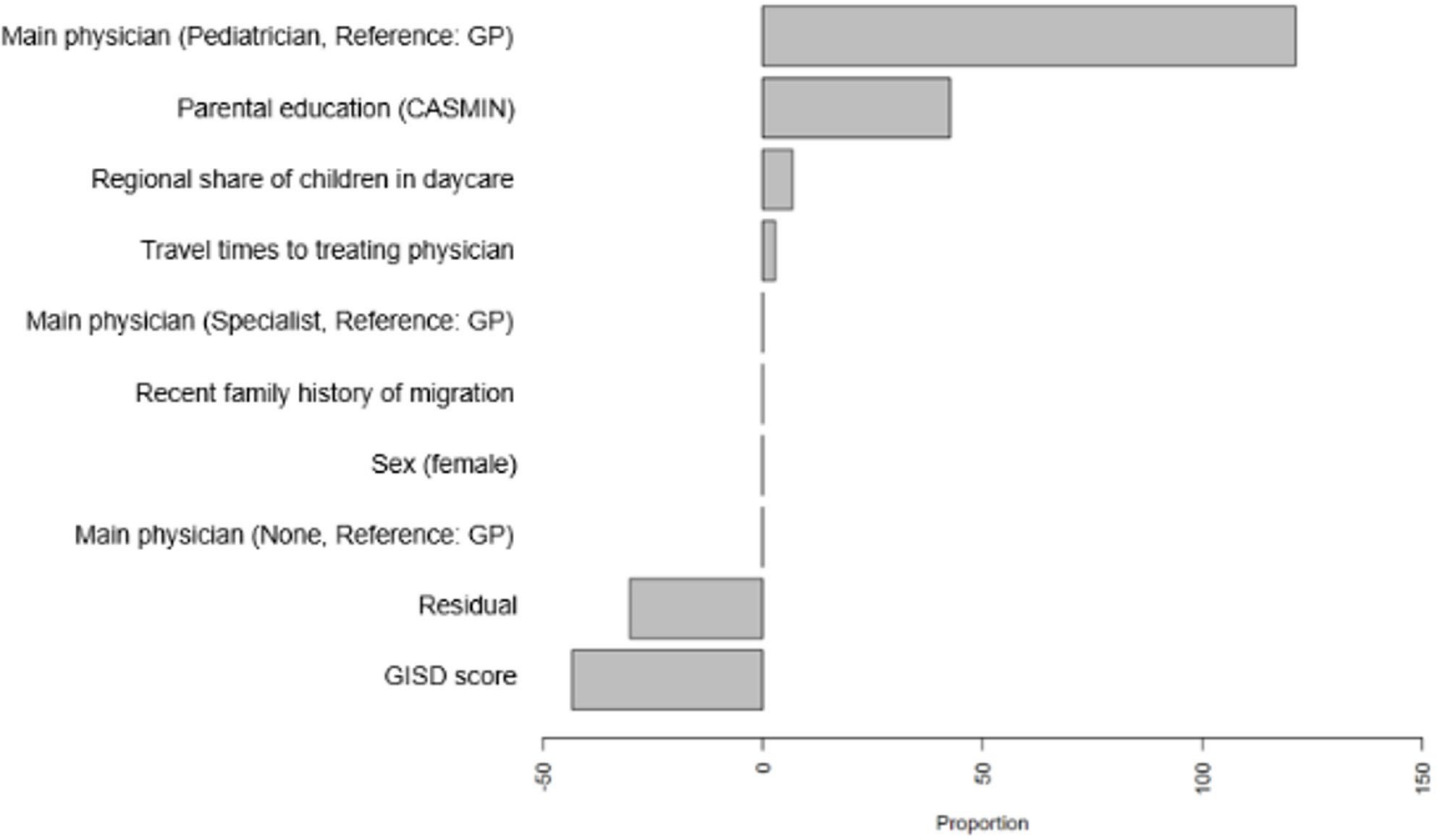
Decomposition

To examine how different factors related to the observed socioeconomic inequality in full vaccination, we decomposed the CI into percentage contributions (Fig. 2). Note that percentage contributions can exceed 100% when some factors contribute in opposing directions and partly offset one another. We excluded place of residence, pediatrician density, and the regional share of children in poverty because they were highly correlated with other covariates. Further details can be found in the Additional file 1: Table S1, Table S2, and Table S3. The largest share of the CI was associated with whether children were primarily seen by a pediatrician rather than a GP (121%). This reflects that children from higher socioeconomic backgrounds are more likely to be seen by pediatricians rather than general practitioners, and that care by pediatricians is associated with higher vaccination uptake. Parental education accounted for a further 43%. Daycare attendance and travel times contributed only small shares, and contributions were less than 1% for a child’s sex, recent family history of migration, and provider type when comparing no HCP contact or predominantly specialist contact with GP-led care. Area-level deprivation (GISD) was associated with higher full vaccination, and because deprivation is more common in lower-SES groups, it reduced pro-higher-SES CI (-43%). The residual component accounted for − 30% of the CI, indicating that the included covariates explain more than the total observed inequality, with offsetting effects captured in the residual.

### Sensitivity analyses

We conducted several sensitivity analyses to examine the robustness of our findings. When we re-estimated the CI using non-vaccination as the outcome, the CI was − 0.037 (95% confidence interval: -0.038 to -0.036), indicating that non-vaccination was more concentrated among lower-SES groups, which is consistent with the small gradient favoring high-SES groups observed for full vaccination. When parental education was used as the ranking variable, the CI was 0.020 (95% confidence interval: 0.019 to 0.021), indicating a similar gradient with slightly smaller magnitude. We also varied the set of covariates included in the decomposition, and the overall pattern of contributions was similar across specifications. For example, when vaccination type (primary series dose vs. booster) was added, it accounted for a large share of the explained component, but this variable does not represent a socioeconomic mechanism. In these models, the contributions attributed to provider types (pediatrician vs. GP), parental education, and area-level deprivation were smaller than in the primary specification but were still among the largest (25%, 3%, and − 10%, respectively). We present the corresponding concentration curves and decomposition results in the Additional file 1: Figure S1, Figure S2, and Figure S3.

## Discussion

We used German statutory health insurance claims data to quantify socioeconomic inequality in childhood vaccination coverage and to examine how different factors contributed to the observed gradient using concentration index decomposition. We found a statistically significant but small socioeconomic gradient in vaccination coverage favoring children from households with higher SES. Provider type accounted for the largest share of this gradient, and parental education also contributed, whereas geographical access measures such as regional travel time to the treating physician explained little of the observed inequality. Given the small magnitude of the concentration index, percentage contributions should be interpreted with caution, as even modest absolute effects can translate into relatively large shares of the overall inequality.

The estimated CI of 0.024 (95% confidence interval: 0.023 to 0.025) places the magnitude of this gradient at the lower end of values reported in the literature (approximately 0.02 to 0.27). Many published estimates, however, come from other outcomes [38, 42, 49, 61, 72–76]. Estimates for pediatric vaccination have been higher (0.20 to 0.27), but also stem from different settings (e.g., low- and middle-income countries) [45, 50] or may reflect differences in how timeliness and adherence to the recommended administration windows are defined and measured (e.g., based on vaccination status at a specific age such as nine months) [51]. Because delayed vaccination is more common in lower-SES groups, the operational definition of timeliness can affect the size of the measured gradient [57]. In the context of Germany’s relatively high vaccination coverage, small CI values are plausible. In Germany, regular well-child visits and their high uptake may support high overall vaccination coverage and narrower socioeconomic disparities [77]. Even so, differences by socioeconomic status persist despite national recommendations and strong evidence on the effectiveness of childhood vaccines [31, 78, 79]. Although the concentration index is small, the absolute difference in vaccination coverage between occupational groups remains non-negligible (approximately 6% points between the lowest and highest groups), indicating that socioeconomic differences, while modest, may still be relevant from a policy perspective.

The contribution of parental education and travel time differed across model specifications. In models that did not adjust for vaccination type (primary-series doses vs. booster doses), parental education contributed 43% of the observed socioeconomic inequality in full and timely vaccination. However, when vaccination type was included, this contribution decreased to 3%. This pattern is consistent with evidence that education-related differences in child immunization tend to be smaller in high-income settings: in a systematic review and meta-analysis, Forshaw et al. (2017) reported a positive association between maternal education and completion of routine child immunization overall, but the association was comparatively weak in Europe, with many European studies reporting no clear relationship [54]. A study from Germany focusing on migrant populations even reported a higher risk of non-vaccination for measles, mumps, rubella, or hepatitis B among children of more highly educated parents [80]. In contrast, travel time contributed less than 3% in all of our specifications, suggesting that geographic accessibility, as captured by this measure, contributes little to the observed socioeconomic gradient. This is consistent with evidence that concerns and attitudes about vaccination can outweigh logistical factors for uptake decisions [81]. At the same time, short travel times do not rule out barriers to access such as limited appointment availability or practices not accepting new patients, which this measure does not capture. Overall, parental education and travel time accounted for only a small share of the observed socioeconomic inequality in vaccination coverage, particularly once differences between primary-series and booster vaccination were taken into account.

In addition to the main findings, several patterns help situate our results within the wider landscape of vaccination research. The type of clinician with whom children had the most outpatient contact accounted for the largest share of the socioeconomic gradient in full vaccination: being seen primarily by a pediatrician rather than a GP contributed 25% even in models adjusted for vaccination type (primary-series doses vs. booster doses). This result extends the district-level findings of Eichner et al. (2017), who reported higher rates of non-vaccination or under-vaccination for measles among children seen by GPs, to a nationwide setting and a broader set of recommended childhood vaccinations. One possible explanation is that pediatricians are more familiar with childhood vaccination schedules and may maintain higher or broader stocks of pediatric vaccines [66]. Another possibility is that appointment availability may differ between provider types: if pediatric practices are not accepting new patients, some families may seek care instead from GPs, who may also have limited capacity. However, this contribution should be interpreted with caution, as provider type may lie on the causal pathway between SES and vaccination, reflect parental selection into different types of care, or capture unobserved preferences.

In reduced models, area-level deprivation (GISD) showed a positive association with full vaccination. However, as our study examined correlations, these findings cannot be interpreted causally, and they may reflect the limitations of using area-level measures to capture individual circumstances. Lastly, a recent family history of migration did not contribute meaningfully to the socioeconomic gradient in the decomposition, suggesting that migration status alone does not capture cultural and behavioral factors relevant to immunization behavior.

To our knowledge, this is the first study to quantify and decompose socioeconomic inequality in childhood vaccination coverage in Germany using health insurance claims data. This contribution complements existing international evidence, which has focused on survey-based data or on low- and middle-income settings. By using large-scale administrative claims data from a high-income country with near-universal coverage, our study provides a population-level perspective on vaccination inequality and its underlying factors in a context where financial barriers are minimal.

Several limitations should nevertheless be acknowledged. First, because only year of birth was available, we could not determine with precision whether vaccinations occurred within the recommended administration windows. In addition, we could not identify the specific vaccine product when multiple products were available for the same disease. This created some uncertainty when distinguishing full from partial vaccination status when schedules differed (e.g., two vs. three required doses); in such cases, we applied the lower dose threshold in our primary definition. Second, the data set did not include a direct measure of income or wealth. Our socioeconomic ranking therefore relied on categorical indicators of occupation type from the policy-holding parent through whom the child was insured. Thirdly, the non-negligible residual component indicates that a substantial share of the observed inequality is not captured by included covariates and may reflect unobserved factors or measurement limitations. More generally, the observed socioeconomic gradient should be interpreted as descriptive rather than causal, as unobserved factors may influence both the SES and vaccination outcomes. Furthermore, because individual- and area-level variables were analyzed within a single-level framework, residual correlation among children residing in the same area was not modelled explicitly. Consequently, the associations observed for area-level covariates should be interpreted as descriptive contextual associations rather than evidence of causal area-level effects. Also, our measure of family history of migration was based on parental citizenship and therefore does not fully capture migration background. Lastly, the analysis was based on data from a single statutory health insurer, which may limit generalizability to the full German population and to other health systems (see also reference blinded for review). Despite these limitations, the large sample and the setting of universal vaccination coverage strengthen the validity and relevance of our findings, as the observed differences persisted even under these conditions.

## Conclusions

Using German health insurance claims data and concentration index decomposition, we found that full and timely vaccination remained below national targets in our sample: only about 80% of recommended immunizations were completed within the recommended time frame, implying that about 20% were incomplete and/or delayed. We also observed a small socioeconomic gradient in full and timely vaccination, favoring children from more advantaged households. In the decomposition, travel time to the treating physician contributed little to the observed inequality, whereas the type of clinician with whom children had the most outpatient contact accounted for a substantial share, with lower completion and timeliness among children primarily seen by general practitioners than among those primarily seen by pediatricians. Parental education contributed to the CI in some specifications, but its share of the CI was sensitive to adjustment for vaccination type (primary-series doses vs. booster doses). Overall, these findings suggest that improving how vaccinations are delivered in settings where children are primarily seen by non-pediatricians may be more relevant for narrowing the observed gradient than seeking further reductions in travel time. The underlying mechanisms remain unclear and warrant further investigation.

## Supplementary Information

Below is the link to the electronic supplementary material.

Supplementary Material 1: Additional file 1: Section 1–Section 4, Table S1–Table S3, Figure S1–Figure S3.

## Data Availability

The data that support the findings of this study are available from the statutory health insurance Techniker Krankenkasse but restrictions apply to the availability of these data, which were used under license for the current study, and thus are not publicly available. Data are, however, available from the authors upon reasonable request and with permission of Techniker Krankenkasse and its supervisory authority.

## Abbreviations

CASMIN: Comparative Analysis of Social Mobility in Industrial Nations
CI: Concentration index
EU: European Union
GISD: German Index of Socioeconomic Deprivation
GP: General practitioner
HCP: Health care provider
pp: Percentage points
RKI: Robert-Koch-Institute
SES: Socioeconomic status
STIKO: Standing Committee on Vaccination
TK: Techniker Krankenkasse (German statutory health insurer)
US: United States
