# Supplementary material for: Socioeconomic inequality in recommended childhood vaccinations in Germany: evidence from insurance claims and concentration index decomposition
Source: BMC Med. 2026 Aug 4;24:423. doi: 10.1186/s12916-026-05113-2 (PMC13445900; doi:10.1186/s12916-026-05113-2)
Supplement: Supplementary file 1 [file 12916_2026_5113_MOESM1_ESM.docx]

**Supplemental material: Socioeconomic inequality in recommended childhood vaccinations in Germany: evidence from insurance claims and concentration index decomposition**

1. **Sample selection and missing data**

We applied several exclusion criteria to define the final study population. First, 1,890 observations were excluded because children were living abroad during the observation period. Second, 110,313 observations were excluded due to incomplete quarterly claims data, which also includes children who died during the observation period. Third, 172,858 observations were excluded due to missing information on parental socioeconomic status, specifically parental occupation or education.

The remaining observations constitute the analytical sample used in the main analyses.

2. **Operationalization of vaccinations and other healthcare events in routine data**

In the following, we present the codes to extract the relevant procedures in German insurance claims data:

- Vaccinations (number of doses for full immunization):
  - Rotavirus (2) :89127, 89127A, 89127B
  - Tetanus (4*/1 for booster): 89124, 89124A, 89124B, 89124R, 89200, 89200A, 89200B, 89201, 89201A, 89201B, 89201R, 89300, 89300A, 89300B, 89302, 89302R, 89303, 89303R, 89303Y, 89400, 89400R, 89500, 89500A, 89500B, 89600, 89600A, 89600B
  - Diphteria (4*/1 for booster): 89100, 89100A, 89100B, 89100R, 89101, 89101A, 89101B, 89101R, 89200, 89200A, 89200B, 89201, 89201A, 89201B, 89201R, 89300, 89300A, 89300B, 89302, 89302R, 89303, 89303R, 89303Y, 89400, 89400R, 89500, 89500A, 89500B, 89600, 89600A, 89600B
  - Pertussis (4*/1 for booster): 89116, 89116A, 89116B, 89116R, 89117, 89117A, 89117B, 89300, 89300A, 89300B, 89303, 89303R, 89303Y, 89400, 89400R, 89500, 89500A, 89500B, 89600, 89600A, 89600B
  - *Haemophilus influenzae* type B (3): 89103, 89103A, 89103B, 89104, 89104A, 89104B, 89203, 89203A, 89203B, 89500, 89500A, 89500B, 89600, 89600A, 89600B
  - Poliomyelitis (3): 89121, 89121A, 89121B, 89121R, 89122, 89122A, 89122B, 89122R, 89122V, 89122W, 89122X, 89302, 89302R, 89400, 89400R, 89500, 89500A, 89500B, 89600, 89600A, 89600B
  - Hepatitis B (3): 89106, 89106A, 89106B, 89107, 89107A, 89107B, 89107R, 89107V, 89107W, 89107X, 89108, 89108A, 89108B, 89108R, 89202, 89202A, 89202B, 89202S, 89202V, 89202W, 89600, 89600A, 89600B
  - Pneumococcal disease (3): 89118, 89118A, 89118B, 89119, 89120, 89120R, 89120V, 89120X
  - Meningococcus C (1): 89114, 89114S, 89115, 89115A, 89115B, 89115R, 89115V, 89115W, 89115X
  - Measles (2): 89113, 89113A, 89113B, 89113Y, 89301, 89301A, 89301B, 89301Y, 89401, 89401A, 89401B
  - Mumps (2): 89301, 89301A, 89301B, 89301Y, 89401, 89401A, 89401B
  - Rubella (2): 89123, 89301, 89301A, 89301B, 89301Y, 89401, 89401A, 89401B
  - Varicella (2): 89125, 89125A, 89125B, 89126, 89126A, 89126B, 89126V, 89126W, 89401, 89401A, 89401B

*The recommendation to move from 3+1 to 2+1 vaccination schedules for base immunization against diphteria, tetanus, pertussis, poliomyelitis, *Haemophilus influenzae* type B and hepatitis B was only issued in mid-2020 after the end of our observation window. *(RKI (2020): STIKO-Empfehlung zur Grundimmunisierung mit dem 6-fach-Impfstoff DTaP-IPV-Hib-HepB im Säuglingsalter ­ nach dem ­2+1-Impfschema. In Epidemiologisches Bulletin 26, pp. 3–24.)*

In the following, we present the operationalization of the CASMIN levels based on German health insurance claims data, which include the 'Tätigkeitsschlüssel' (occupation code) provided by the Federal Employment Agency. This occupation code also contains information on basic and higher education as well as vocational training.

- CASMIN 1a: no school-leaving qualification
  - Education: Level 1 (Inadequately completed general elementary education) or Level 9 (Unknown)
  - Vocational training: Level 1 (Without vocational training) or Level 9 (Unknown)
  - Combinations of Level 9 and Level 9 lead to the exclusion of observations
- CASMIN 1b: lower secondary school certificate without vocational training
  - Education: Level 2 (Lower secondary education)
  - Vocational training: Level 1 (Without vocational training) or Level 9 (Unknown)
- CASMIN 1c: lower secondary school certificate with vocational training
  - Education: Level 1 (Inadequately completed general elementary education), Level 2 (Lower secondary education) or Level 9 (Unknown)
  - Vocational training: Level 2 (Vocational training / apprenticeship) or Level 3 (Master craftsman / technician)
- CASMIN 2b: intermediate school certificate without vocational training
  - Education: Level 3 (Intermediate secondary education)
  - Vocational training: Level 1 (Without vocational training) or Level 9 (Unknown)
- CASMIN 2a: intermediate school certificate with vocational training
  - Education: Level 3 (Intermediate secondary education)
  - Vocational training: Level 2 (Vocational training / apprenticeship) or Level 3 (Master craftsman / technician)
- CASMIN 2c_generic: higher education entrance qualification without vocational training
  - Education: Level 4 (Higher secondary education)
  - Vocational training: Level 1 (Without vocational training) or Level 9 (Unknown)
- CASMIN 2c_vocational: higher education entrance qualification with vocational training
  - Education: Level 4 (Higher secondary education)
  - Vocational training: Level 2 (Vocational training / apprenticeship) or Level 3 (Master craftsman / technician)
- CASMIN 3a: university of applied sciences degree
  - Education: Any level
  - Vocational training:Level 4 (Bachelor’s degree or equivalent)
- CASMIN 3b: university degree
  - Education: Any level
  - Vocational training: Level 5 (Advanced university degree: Diplom, Magister, Master, State exam)
- CASMIN 3c: doctorate/PhD
  - Education: Any level
  - Vocational training: Level 6 (Doctorate / PhD)

3. **Concentration index and decomposition equations**

The concentration index (CI) can be expressed as:

$$CI=\frac{2}{\mu}\mathrm{Cov}(y,R),$$

where $y$ is the outcome variable, $\mu$ its mean, and $R$ the fractional rank of individuals in the socioeconomic distribution.

Because vaccination status is a bounded binary variable, we use the Erreygers-corrected concentration index:

$$E=\frac{4\mu}{b-a}\cdot CI,$$

where $a$ and $b$ denote the lower and upper bounds of the outcome variable. For binary outcomes bounded between 0 and 1, this simplifies to:

$$E=4\mu\cdot CI,$$

which rescales the CI to account for the bounds of the outcome variable (Erreygers 2009).

Following standard approaches, the concentration index can be decomposed as:

$$CI=\sum_{k} \left( \frac{\beta_{k}\bar{x}_{k}}{\mu} \right)CI_{k}+\frac{G_{\varepsilon}}{\mu},$$

where $\beta_{k}$ denotes the association between covariate $x_{k}$ and the outcome, $\bar{x}_{k}$ the mean of $x_{k}$, $CI_{k}$ the concentration index of $x_{k}$, and $G_{\varepsilon}$ the generalized concentration index of the residual term.

For nonlinear models such as logistic regression, this decomposition relies on a linear approximation. In our analysis, we used coefficients from a generalized linear model with a logit link together with predicted outcome values to approximate the decomposition, which is standard in applied studies of inequality decomposition for binary outcomes (Debie et al., 2010, Doherty et al. 2014).

The resulting decomposition should therefore be interpreted as an approximation of the contribution of covariates to inequality and is not numerically identical to a direct decomposition of the Erreygers-corrected concentration index.

The residual component was calculated as:

$$\frac{G_{\varepsilon}}{\mu}=CI-\sum_{k} \left( \frac{\beta_{k}\bar{x}_{k}}{\mu} \right)CI_{k}.$$

4. **Correlation matrix**

The correlation matrix of the variables included in the analysis is presented below.

|  | **Child poverty** | **Travel times** | **Base immunization** | **Deprivation index (GISD)** | **Place of residence: urban** | **Place of residence: Eastern Germany** | **Daycare attendance** | **Pediatrician density** | **Parental education** | **Parental occupation** |
| --- | --- | --- | --- | --- | --- | --- | --- | --- | --- | --- |
| **Child poverty** |  | -0.67 | 0.02 | 0.37 | 0.33 | 0.37 | 0.22 | 0.42 | 0.09 | 0.02 |
| **Travel times** | -0.67 |  | -0.04 | 0.25 | -0.61 | -0.18 | -0.18 | -0.68 | -0.2 | -0.12 |
| **Base immunization** | 0.02 | -0.04 |  | -0.03 | 0.03 | -0.01 | 0 | 0.03 | 0.04 | 0.01 |
| **Deprivation index (GISD)** | 0.37 | 0.25 | -0.03 |  | -0.34 | 0.44 | 0.09 | -0.12 | -0.14 | -0.13 |
| **Place of residence: urban** | 0.33 | -0.61 | 0.03 | -0.34 |  | -0.12 | -0.1 | 0.31 | 0.13 | 0.08 |
| **Place of residence: Eastern Germany** | 0.37 | -0.18 | -0.01 | 0.44 | -0.12 |  | 0.76 | 0.24 | 0.06 | 0.01 |
| **Daycare attendance** | 0.22 | -0.18 | 0 | 0.09 | -0.1 | 0.76 |  | 0.32 | 0.1 | 0.04 |
| **Pediatrician density** | 0.42 | -0.68 | 0.03 | -0.12 | 0.31 | 0.24 | 0.32 |  | 0.17 | 0.11 |
| **Parental education** | 0.09 | -0.2 | 0.04 | -0.14 | 0.13 | 0.06 | 0.1 | 0.17 |  | 0.55 |
| **Parental occupation** | 0.02 | -0.12 | 0.01 | -0.13 | 0.08 | 0.01 | 0.04 | 0.11 | 0.55 |  |

Table S1: Correlation matrix

5. **Regression and decomposition results**

In addition to the main results presented in the manuscript, we report the regression model underlying the decomposition as well as the detailed decomposition results for the primary specification.

We estimated a generalized linear model with a logit link to assess the association between covariates and full and timely vaccination at the person–vaccine level.

| **Variable** | **Estimate** | **Standard error** | **z-value** | **p-value** |
| --- | --- | --- | --- | --- |
| **Intercept** | 0.211 | 0.007 | 28.96 | <0.001 |
| **Sex (female)** | 0.016 | 0.003 | 4.97 | <0.001 |
| **Recent family history of migration** | 0.004 | 0.006 | 0.75 | 0.452 |
| **Parental education (CASMIN)** | 0.005 | 0.001 | 6.93 | <0.001 |
| **Regional share of children in daycare** | 0.041 | 0.002 | 24.74 | <0.001 |
| **Deprivation score (GISD)** | 0.070 | 0.002 | 41.98 | <0.001 |
| **Travel time to treating physician** | -0.007 | 0.002 | -3.74 | <0.001 |
| **Main physician (None, Reference: GP)** | -13.80 | 10.01 | -1.38 | 0.168 |
| **Main physician (Pediatrician, Reference: GP)** | 1.222 | 0.006 | 216.81 | <0.001 |
| **Main physician (Specialist, Reference: GP)** | 0.784 | 0.022 | 36.15 | <0.001 |

Table S2: Logistic regression model for full and timely vaccination

We decomposed the concentration index into percentage contributions, absolute contributions, elasticities, and concentration indices of the covariates based on the regression model.

| **Variable** | **Contribution (%)** | **Contribution (Abs)** | **Elasticity** | **CIₖ** |
| --- | --- | --- | --- | --- |
| **Residual** | -30.20 | -0.00050 | — | — |
| **Sex (female)** | -0.04 | -0.00000 | 0.00051 | -0.00121 |
| **Recent family history of migration** | 0.01 | 0.00000 | 0.00002 | 0.00558 |
| **Parental education (CASMIN)** | 42.68 | 0.00071 | 0.00219 | 0.32319 |
| **Regional share of children in daycare** | 6.77 | 0.00011 | 0.00588 | 0.01913 |
| **Deprivation score (GISD)** | -43.26 | -0.00072 | 0.01235 | -0.05816 |
| **Travel time to treating physician** | 2.86 | 0.00005 | -0.00059 | -0.07979 |
| **Main physician (None, Reference: GP)** | -0.04 | -0.00000 | -0.00104 | 0.00061 |
| **Main physician (Pediatrician, Reference: GP)** | 121.18 | 0.00201 | 0.07574 | 0.02657 |
| **Main physician (Specialist, Reference: GP)** | 0.03 | 0.00000 | 0.00024 | 0.00221 |

Table S3: Decomposition of the concentration index for full and timely vaccination
Note: Contributions may exceed 100% when factors contribute in opposing directions and partly offset one another. The residual represents the unexplained component of inequality. Contributions are interpreted in terms of their relative importance rather than statistical significance. The concentration indices of the covariates (CIₖ) and the overall index reported within the decomposition are based on the model-based approximation implemented in the rineq R package and are therefore not directly comparable to the standalone Erreygers-corrected concentration index reported in the main text.

6. **Sensitivity analyses**

In this section, we present additional results from the sensitivity analyses. The first figure S1 shows the concentration curves when non-vaccination was used as the outcome instead of full vaccination. The second figure S2 presents the concentration curves when parental education was used as the ranking variable instead of parental occupation.

Figure S1: Concentration curve (non-vaccination as outcome variable)

Figure S2: Concentration curve (parental education as socioeconomic ranking variable)

We further report the results of the decomposition analysis when vaccination type (primary series dose vs. booster) was included as an additional covariate (Figure S3).

Figure S3: Decomposition including immunization type (base vs. booster vaccination as covariate)
